# Identification of CD8^+^ T cell subsets that normalize in early-treated people living with HIV receiving antiretroviral therapy

**DOI:** 10.1186/s12981-022-00465-0

**Published:** 2022-09-14

**Authors:** Federico Perdomo-Celis, David Arcia-Anaya, Juan Carlos Alzate, Paula A. Velilla, Francisco J. Díaz, Maria Paulina Posada, María T. Rugeles, Natalia A. Taborda

**Affiliations:** 1 Grupo Inmunovirología, Facultad de Medicina, Universidad de Antioquia - UdeA, Medellín, Colombia; 2 Centre for Cancer Immunology, Faculty of Medicine, University Hospital Southampton, Southampton, UK; 3 Unidad de Investigación Clínica - Corporación para Investigaciones Biológicas, Medellín, Colombia; 4 IPS SIES Salud, Medellín, Colombia; 5 Grupo de Investigaciones Biomédicas Uniremington, Programa de Medicina, Facultad de Ciencias de la Salud, Corporación Universitaria Remington, Medellín, Colombia

**Keywords:** HIV, CD8-positive T-lymphocytes, Antiretroviral therapy, Immune reconstitution

## Abstract

**Background:**

Although combined antiretroviral therapy (cART) has decreased the mortality associated with HIV infection, complete immune reconstitution is not achieved despite viral suppression. Alterations of CD8^+^ T cells and some of their subpopulations, such as interleukin (IL)-17-producing cells, are evidenced in treated individuals and are associated with systemic inflammation and adverse disease outcomes. We sought to evaluate if different CD8^+^ T cell subsets are differentially normalized during a clinical follow-up of people living with HIV (PLWH) receiving suppressive cART.

**Methods:**

We explored the changes in the frequencies, activation/exhaustion phenotypes (HLA-DR, CD38, PD-1, and TIM-3), and function (total and HIV-specific cells expressing CD107a, perforin, granzyme B, interferon [IFN]-γ and IL-17) of CD8^+^ T cells from early-treated PLWH receiving cART in a 1-year follow-up, using a multidimensional flow cytometry approach.

**Results:**

Despite continuous cART-induced viral suppression and recovery of CD4^+^ T cells, after a 1-year follow-up, the CD8^+^ T cell counts, CD4:CD8 ratio, PD-1 expression, and IL-17 production by CD8^+^ T cells exhibited incomplete normalization compared with seronegative controls. However, the proportion of CD8^+^ T cells with an exhausted phenotype (co-expressing PD-1 andTIM-3), and cells co-expressing cytotoxic molecules (Perforin and Granzyme B), reached normalization.

**Conclusions:**

Although suppressive cART achieves normalization of CD4^+^ T cell counts, only particular subsets of CD8^+^ T cells are more rapidly normalized in PLWH receiving cART, which could be routinely used as biomarkers for therapy efficiency in these patients.

**Supplementary Information:**

The online version contains supplementary material available at 10.1186/s12981-022-00465-0.

## Background

In the combined antiretroviral therapy (cART) era, extensive efforts have been made to reach the reconstitution of immune parameters in people living with HIV (PLWH). It has been previously demonstrated that a poor reconstitution of immune cell populations has been associated with an increased risk of AIDS progression in patients receiving cART [1, 2]. Nonetheless, despite continuous cART-induced viral suppression, dysfunction of several immune cells, including CD8^+^ T cells, is observed due to the persistent immune activation, leading to an exhaustion state [3–7]. Overexpression of the activation markers HLA-DR and CD38 and the exhaustion markers Programmed Death (PD)-1 and T cell immunoglobulin and mucin-domain containing-3 (TIM-3) is found in CD8^+^ T cells from PLWH. The expression of these markers has been associated with an altered cytotoxic program, low cytokine production, and impaired cell proliferation and survival [8–11]. Although these activation/exhaustion phenotypes are observed in the total CD8^+^ T cell cells, specific subpopulations of these cells, such as interleukin (IL)-17-producing (Tc17) cells, are differentially affected by the persistent activation and inflammatory state during HIV infection [10, 12]. Despite this, the effect of continuous cART-induced viral suppression on the reconstitution of different CD8^+^ T cell populations is yet to be defined.

This study aimed to evaluate longitudinally the changes in the frequency, phenotype, and function of total CD8^+^ T cells in PLWH receiving cART in Medellín, Colombia. Using a flow cytometry data-based predictive model, we identified CD8^+^ T cell clusters reaching normalization as an effect of continuous therapy. Changes in HLA-DR, CD38, the effector molecules granzyme B, perforin, CD107a, and IFN-γ distinguished cell clusters reaching immune reconstitution, which can be used as a straightforward marker of therapeutic success in PLWH.

## Materials and methods

### Study design, donors, and samples

Two groups of individuals were recruited between December 2016 to August 2019: (i) PLWH receiving cART who attended HIV health care programs in Medellín, Colombia, and (ii) seronegative individuals. The inclusion criteria for PLWH were as follows: (i) Receiving cART for more than 1 year, with a viral load < 20 HIV RNA copies/mL at the time of sampling; (ii) Reached this level of viral load in less than 26 weeks of treatment; (iii) Received only one therapeutic scheme; (iv) CD4^+^ T cell counts between 500 cells/µL and 1000 cells/µL, (v) Absence of comedications, smoking or use of other substances; and (vi) Absence of previous therapeutic failure (sustained increase in viral load or decrease of CD4^+^ T cell counts despite therapy), AIDS-defining diseases or non-AIDS conditions, or clinically evident coinfections after cART initiation. During the first visit, a clinical and laboratory evaluation was performed (sample 1). Then, a follow-up was performed 1 year after the first sampling moment to detect any increase in viral load, decrease in CD4^+^ T cell counts, or the development of AIDS-related or non-AIDS conditions (sample 2) [13]. In both sampling moments, 10 mL of venous blood were collected from each patient in ethylenediaminetetraacetic acid (EDTA)-containing tubes, and the phenotyping of circulating T cells was performed immediately. A fraction of the blood was centrifuged at 300×*g*; plasma was used to determine viral load with the approved clinical diagnostic test RT-PCR Ampliprep-Cobas (Roche, Basel, Switzerland), with a detection limit of 20 copies/mL; the cellular fraction was used to isolate peripheral blood mononuclear cells (PBMC) via density gradient centrifugation with Ficoll-Paque (Ficoll Histopaque-1077; Sigma-Aldrich, St. Louis, MO). Notably, samples from seronegative controls were included at both timepoints to corroborate that changes in CD8^+^ T cell populations were specific to PLWH and not a product of technical variability or physiological changes.

### Phenotyping of circulating CD8^+^ T cells

The phenotype of CD8^+^ T cells in peripheral blood was evaluated by flow cytometry, as previously described (Additional file 1: Figure S1) [9, 10]. Antibodies used for flow cytometry analyses are listed in Additional file 7: Table S1. Cells were acquired on an LSR Fortessa cytometer (BD, Franklin Lakes, NJ, USA), using the FACS Diva software v.6.0, within 1 h of completing the staining. At least 25,000 CD3^+^ CD8^+^ events were acquired. Data were analyzed using FlowJo Software version 10.5 (Tree Star, Inc, Ashland, OR, USA). Fluorescence minus one (FMO) controls were included to define positive thresholds.

### Functional analysis of CD8^+^ T cells

Polyclonal stimulation of PBMC was performed with PMA-ionomycin, and antigen-specific responses were evaluated after stimulation with a pool of HIV-1 subtype B consensus Gag peptides, as previously described [9, 10]. After stimulation, intracellular cytokine staining was performed using flow cytometry antibodies described in Additional file 7: Table S1, using an LSR Fortessa cytometer (BD).

### CITRUS analysis

Hierarchical clustering using Ward’s linkage and Euclidean distance was performed with the CITRUS algorithm (cluster identification, characterization, and regression) [14], using the R package found at https://github.com/nolanlab/citrus, which converts multidimensional single-cell data to a hierarchy of related clusters based on the expression of cell markers. First, cells were manually gated using FlowJo v10.5.0 (Tree Star, Ashland, OR, USA), and the CD8^+^ T cell population was exported as an FSC file. Then, the FSC files were imported into the CITRUS R package, and 2000 events were analyzed from each sample for clustering. Each sample was assigned to a group (i. e. HIV sample 1, HIV sample 2, and seronegative controls), and CD8^+^ T cells were clustered based on the expression and abundance of HLA-DR, CD38, CD107a, IL-17, CCL5, IFN-γ, granzyme B, and perforin. The correlative model SAM (Significance Analysis of Microarrays) and the predictive model PAM (Predictive Analysis of Microarrays) were performed with comparable cluster results. Of note, SAM detects subsets of cluster properties correlated with the experimental endpoint (i.e., the group to which each individual belongs) but are not necessarily accurate predictors of the outcome. In contrast, PAMR establishes a model to identify subsets of cluster properties that are the best predictors of the experimental endpoint. Clusters containing at least 5% of all clustered cells were graphically displayed. Cluster plots depict the clustering hierarchy, and nodes are scaled based on the frequency of cells in that cluster. The analyses of the set of samples were independently run at least five times, with comparable results among them.

### Statistical analysis

Data are presented as medians and ranges. The Mann–Whitney and Wilcoxon tests were used to compare two independent or paired data, respectively. In all cases, a P-value < 0.05 was considered significant. The GraphPad Prism software v.8.0 (GraphPad Software, La Jolla, CA, USA) was used for the statistical analysis. CD8^+^ T cell features in PLWH reaching levels similar to those in seronegative individuals (non-significant P-value between both groups) after the 1-year follow-up were considered as reaching normalization.

## Results

### Partial reconstitution of CD8^+^ T cells in PLWH despite cART

Among the cohort of PLWH, the majority were diagnosed at stage 2 (13–81.25%), while only one individual was diagnosed at stage 1 (6.25%), and two at stage 3 (12.5%) [15]. At the time of enrollment, PLWH had a median (IQR) of 36 (25–100) months since HIV diagnosis, with 29 (21–72) months of therapy. All patients initiated treatment during chronic infection with a combination of two Nucleoside Reverse Transcriptase Inhibitors (NRTIs), and either one Non-nucleoside Reverse Transcriptase Inhibitors (NNRTIs) or one Integrase Strand Transfer Inhibitors (INSTI) (Additional file 8: Table S2). First, we evaluated the CD4^+^ and CD8^+^ T cell counts and the CD4:CD8 ratio in seronegative individuals and PLWH. CD4^+^ T cell counts in PLWH were significantly decreased at the first sampling in PLWH, but after a 1-year follow-up these individuals showed a significant recovery in CD4^+^ T cell counts. Nonetheless, CD8^+^ T cell counts remained significantly increased at both sampling moments, along with a low CD4:CD8 ratio (Table 1). No individuals from the PLWH group developed therapeutic failure or clinically evident coinfections during follow-up.

**Table 1:** Demographic data and T cell counts in the study cohort

Parameter	HIV sample 1 Median (IQR)	HIV sample 2 Median (IQR)	Seronegative Median (IQR)	P-value HIV sample 1 vs HIV sample 2^1^	P-value HIV sample 1 vs. seronegative^2^	P-value HIV sample 2 vs. seronegative^2^
n	16		15	NA	NA	NA
Age (years)	33 (24–52)		27 (25–28)	NA	0.16	NA
Estimated date of serodiagnosis (Months after HIV diagnosis)	36 (25–100)		NA	NA	NA	NA
CD4^+^ T cells count (cells/μL)	642 (533–705)	854 (811–1122)	634 (535–1192)	0.0007	0.49	0.08
CD8^+^ T cells count (cells/μL)	830 (598–1078)	948 (552–1265)	460 (361–537)	0.27	< 0.0001	0.0007
CD4:CD8 ratio	0.75 (0.59–0.99)	1.15 (0.72–1.58)	1.6 (1.4–2.3)	0.0037	< 0.0001	0.0163

^1^Wilcoxon test

^2^Mann–Whitney test. NA: Does not apply; IQR: interquartile range

### CITRUS identifies a particular cluster of CD8^+^ T cells reaching normalization

**Fig. 1:**
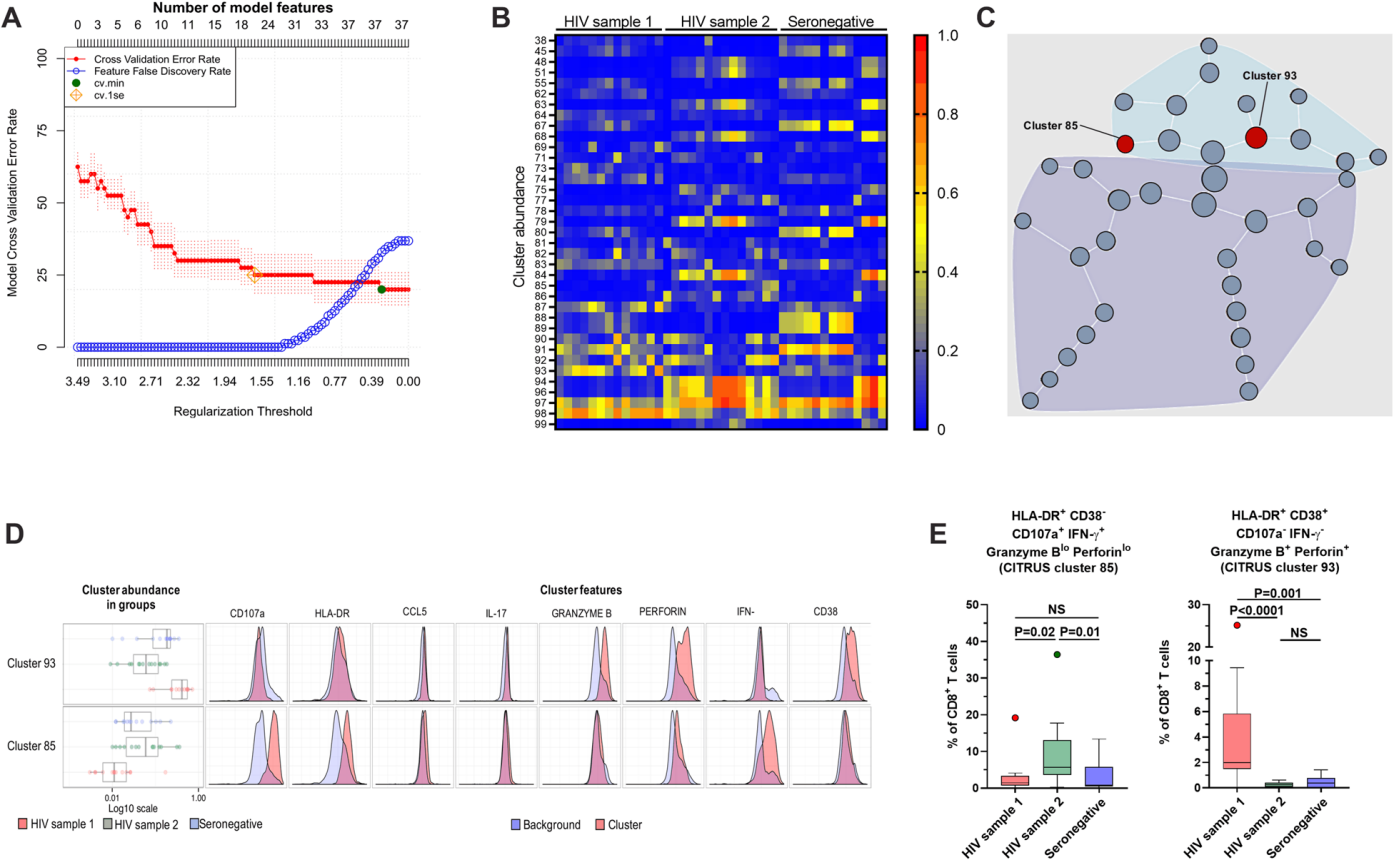
CD8^+^ T cell functional features as markers of immune reconstitution in PLWH receiving cART. **A** PAMR estimated model accuracy and feature false discovery rate as a function of model regularization threshold from CITRUS algorithm. The green point indicates the threshold and error rate of the selected model. **B** Heat map showing the abundance of each cluster obtained with the model in the three groups of individuals included (HIV samples 1 and 2, and seronegative individuals’ sample 1). **C** Relationship of the clusters obtained with the model. The two discriminatory clusters (93 and 85) are shown in red. **D** Abundance and features of the two selected discriminatory clusters (93 and 85) in the three groups of individuals included (HIV samples 1 and 2, and seronegative individuals’ sample 1). **E** Frequencies of cluster 85 (left panel) and cluster 93 (right panel) obtained by manual flow gating in the three group of samples. P value of Dunn’s post-hoc test

To explore which CD8^+^ T cell populations were preferentially reconstituted in PLWH, we performed hierarchical cell clustering in an unsupervised manner using the CITRUS algorithm by analyzing the functional capacity and activation phenotypes of CD8^+^ T cells after polyclonal stimulation with PMA/ionomycin. The performance of the PAMR model (estimated model accuracy and feature False Discovery Rate) is shown in Fig. 1A. The model with the lowest cross-validation error rate and false discovery rate was chosen (cv min; green point in Fig. 1A). Next, the cell clusters obtained with the PAMR predictive model were analyzed for abundance in the three groups of individuals included (HIV sample 1, HIV sample 2, and seronegative controls). Figure 1B and C show the abundance of the cell clusters obtained and their relationships, respectively. Two clusters (93 and 85) were chosen based on the changes after a 1-year follow-up in PLWH, reaching similar levels to those in seronegative individuals (Fig. 1D). Cluster 93 includes cells characterized by a low expression of CD107a and IFN-γ, and high expression of CD38, perforin, and granzyme B respective to the background. This cluster is increased in PLWH sample 1 but decreases to levels similar to those in seronegative individuals after a 1-year follow-up (HIV sample 2) (Fig. 1E, right). On the other hand, cluster 85 includes cells characterized by a high expression of CD107a, IFN-γ, and HLA-DR, and low expression of CD38, perforin, and granzyme B. This cluster is decreased in PLWH sample 1 but increases to levels similar to those in seronegative individuals after a 1-year follow-up (HIV sample 2) (Fig. 1E, left).

### Preferential reconstitution of effector and exhausted phenotypes of CD8^+^ T cells

**Fig. 2:**
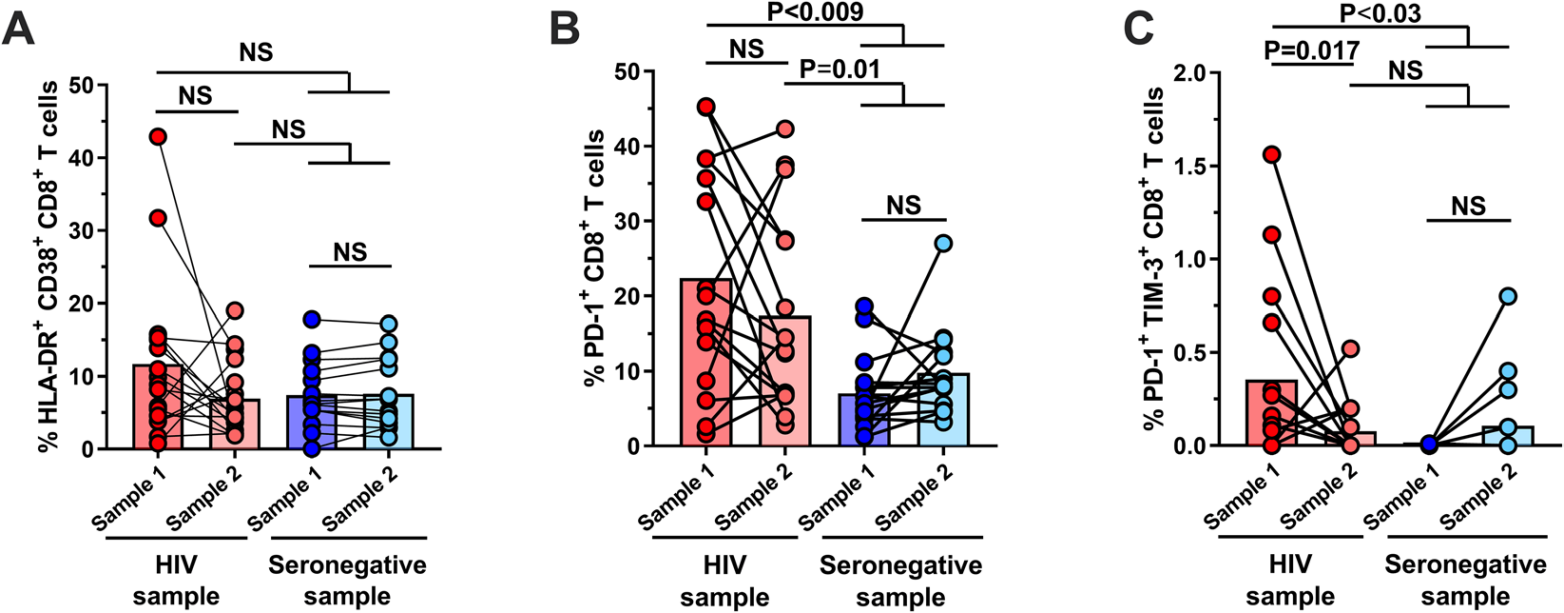
Partial reconstitution of the activation/exhaustion phenotype despite continuous cART in PLWH. Frequencies of CD8^+^ T cells that are HLA-DR^+^ CD38^+^ (**A**), PD-1^+^ (**B**), and PD-1^+^ TIM-3^+^ (**C**) in PLWH and seronegative controls samples 1 and 2. NS: Not statistically significant. Mann–Whitney and Wilcoxon tests were used to analyze independent and paired samples, respectively. HIV samples = 16; Seronegative samples = 15

Considering that the differential expression of activation (HLA-DR and CD38) and effector markers (CD107a, perforin, Granzyme B, and IFNγ) in clusters 93 and 85 were associated with reconstitution in PLWH, we evaluated if CD8^+^ T cell populations with a classically activated phenotype (HLA-DR^+^ CD38^+^) also showed signs of reconstitution in these patients. Furthermore, we compared the levels of exhaustion (PD-1^+^ and PD-1^+^ TIM-3^+^) between PLWH and seronegative individuals, considering that the chronic antigenic stimulation in HIV infection leads to an upregulation of these markers in CD8^+^ T cells [16]. Although HLA-DR^+^ CD38^+^ CD8^+^ T cells were not significantly increased in sample 1 from PLWH, after a 1-year follow up these cells showed a tendency towards normalization, whereas the total PD-1^+^ CD8^+^ T cell population remained unchanged in PLWH (Fig. 2A and B). Nonetheless, the levels of PD-1^+^ TIM-3^+^ CD8^+^ T cells reached levels similar to those in seronegative individuals (Fig. 2C). The normalization pattern of HLA-DR^+^ CD38^+^ CD8^+^ T cells was not associated with a longer time on cART therapy, as the decrease in the percentage of these cells was observed after the 1-year follow-up in patients receiving cART for 1–3 years, or for more than 3 years (Additional file 2: Fig. S2A).

**Fig. 3:**
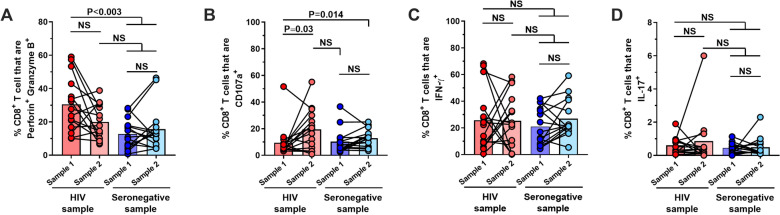
Reconstitution of the cytotoxic program of total CD8^+^ T cells in PLWH receiving cART. Frequencies of CD8^+^ T cells that are Perforin^+^ Granzyme B^+^ (**A**), CD107a^+^ (**B**), IFN-γ^+^ (**C**), and IL-17^+^ (**D**) after stimulation with PMA/ionomycin, in PLWH and seronegative controls samples 1 and 2. NS: Not statistically significant. Mann–Whitney and Wilcoxon tests were used to analyze independent and paired samples, respectively. HIV samples = 16; Seronegative samples = 15

Interestingly, PD-1^+^ CD8^+^ T cells were higher in individuals with a longer time on cART (Additional file 2: Fig. S2B), while the decrease of PD-1^+^ TIM-3^+^ CD8^+^ T cells was mostly observed in PLWH sample 2 compared with sample 1 regardless of the time of cART treatment (Additional file 2: Fig. S2C). Such phenotypical differences between PLWH and seronegative donors were not related to age since we did not observe differences when comparing young versus old PLWH (Additional file 3: Fig. S3A and B). Regarding the functional capacities of polyclonally-stimulated CD8^+^ T cells, we observed that the frequencies of Perforin^+^ Granzyme B^+^ CD8^+^ T cells were altered at the first measurement but reached similar levels to those in seronegative individuals after a 1-year follow-up. In contrast, the degranulation capacity increased at the second sampling in PLWH (Fig. 3A and B). The frequencies of Tc1 and Tc17 cells were comparable between PLWH and seronegative donors at all time points (Fig. 3C and D).

### Tc17 cells are not adequately reconstituted in cART-treated individuals

**Fig. 4:**
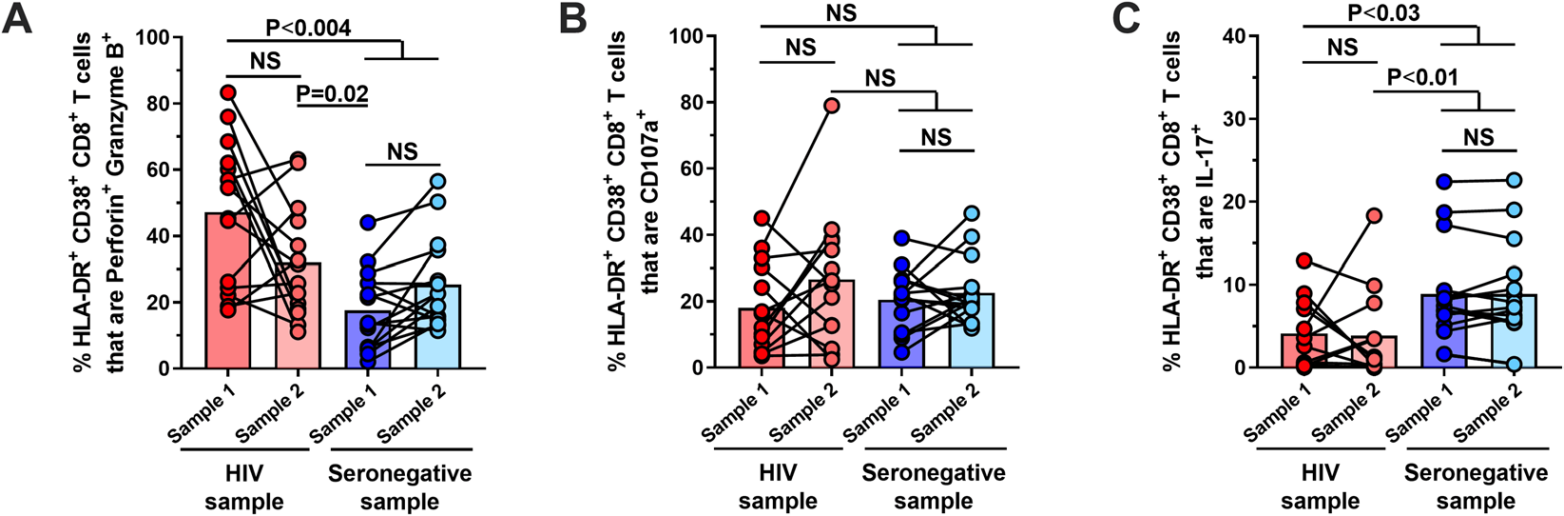
Reconstitution of the cytotoxic program, but not IL-17 production, of HLA-DR^+^ CD38^+^ CD8^+^ T cells in PLWH receiving cART. Frequencies of HLA-DR^+^ CD38^+^ CD8^+^ T cells that are Perforin^+^ Granzyme B^+^ (**A**), CD107a^+^ (**B**), and IL-17^+^ (**C**) after stimulation with PMA/ionomycin, in PLWH and seronegative controls samples 1 and 2. NS: Not statistically significant. Mann–Whitney and Wilcoxon tests were used to analyze independent and paired samples, respectively. HIV samples = 16; Seronegative samples = 15

We then focused our analyses on HLA-DR^+^ CD38^+^ CD8^+^ T cells since they are significant producers of IL-17 [10], express high amounts of cytotoxic molecules corresponding to an effector memory profile [9], and their effector functions are impaired during HIV infection [9, 10, 17]. Like total CD8^+^ T cells, at the first measurement, HLA-DR^+^ CD38^+^ CD8^+^ T cells from PLWH exhibited higher frequencies of Perforin^+^ Granzyme B^+^ cells and lower proportions of CD107a^+^ cells (Fig. 4A and B); however, after the 1-year follow-up, activated CD8^+^ T cells co-expressing Perforin and Granzyme B decreased to levels similar to seronegative individuals, while HLA-DR^+^ CD38^+^ CD8^+^ T cells expressing CD107a showed an increase in the second sample of PLWH, concordant with the data observed in total CD8^+^ T cells (Fig. 4A and B). In contrast, HLA-DR^+^ CD38^+^ Tc17 cells in PLWH remained at significantly lower frequencies than seronegative controls (Fig. 4C). There were no differences in the frequencies of HLA-DR^+^ CD38^+^ Tc1 and CCL5-producing cells between PLWH and seronegative individuals in any of the measurements (Additional file 3: Fig. S4A and B). The frequencies of HIV-specific CD107a^+^ remained unchanged in PLWH after a 1-year follow-up, irrespective of the length of cART therapy (Additional file 5: Fig. S5A). Nonetheless, we observed a significant decrease of HIV-specific Tc1 cells after 1-year follow-up in individuals with 1–3 years of therapy at the time of study inclusion (Additional file 5: Fig. S5B), and a significantly higher proportion of Tc17 cells in the first sampling of individuals with > 3 years of cART (Additional file 5: Fig. S5C). Considering that none of the patients in the present study developed virological therapeutic failure, and the low number of data points in these analyses, further investigations are required in order to elucidate if the length of cART directly influences the reconstitution of Tc1 and Tc17 cells. This finding could be related to the disturbance of this subset in total CD8^+^ T cells. Our previous reports have shown that PLWH receiving cART for more than 25 months has a better reconstitution in HLA-DR^+^ CD38^+^ Tc17 cells than individuals with a shorter length of treatment [9, 10]. Consistently, PLWH with more than 25 months of cART had higher (albeit not statistically significant) frequencies of HLA-DR^+^ CD38^+^ Tc17 cells at both sampling moments (Additional file 4: Fig. S4C), suggesting that longer treatment times are required for the reconstitution of this population. In contrast, we did not observe a correlation between the frequencies of HLA-DR^+^ CD38^+^ Tc17 cells (that have more persistent dysfunction), and the CD4:CD8 ratio (rho = − 0.01, P = 0.9), associated with immune dysfunction in HIV infection [18, 19]. In addition, when we classified PLWH according to the time post-cART (1–3 years vs. > 3 years at the time of study inclusion), we observed that the normalization in HLA-DR^+^ CD38^+^ CD8^+^ T cells that are Perforin^+^ Granzyme B^+^ after 1-year follow-up in PLWH (Fig. 4A) was mainly concentrated in individuals with 1–3 years post-cART (Additional file 6: Fig. S6A).

## Discussion

Here we show that there is limited reconstitution of the CD8^+^ T cell counts, CD4:CD8 ratio, and the levels of activation/exhaustion of circulating CD8^+^ T cells, concomitant with altered effector functions, in PLWH despite continuous suppressive cART. We observed defects in the cytotoxic program and IL-17 production by CD8^+^ T cells but not in IFN-γ and CCL5 production. These disturbances are present despite the recovery of CD4^+^ T cell counts. These results agree with previous reports demonstrating the effectiveness of cART in recovering the CD4^+^ T cell counts in PLWH [20], which is highly related to the suppression of viral replication and with an early start of cART after serodiagnosis [21, 22]. However, the lack of therapy-induced reconstitution of CD8^+^ T cells is associated with the activation/inflammatory burden [18, 23, 24], which is only partially modulated by cART [24–26]. In addition, there was a selective normalization in the activation/exhaustion phenotype and the cytotoxic program of CD8^+^ T cells, but not IL-17 production. Importantly, the fact that Tc1 and Tc17 levels were comparable between PLWH and seronegative individuals at both sampling points, and that IL-17 production has been associated with normalization mainly in CD4^+^ T cells [27], suggests that cART may differentially restore IL-17 production in CD4^+^ T cells, rather than CD8^+^ T cells.

The finding of cluster 93 in the unsupervised clustering analysis is concordant with our previous observations, where the cytotoxic program of CD8^+^ T cells from PLWH is altered in comparison with seronegative individuals, with lower degranulation capacity (expression of CD107a) and higher intracellular expression of granzyme B and perforin. However, no recovery in the frequency of HLA-DR^+^ CD38^+^ CD8^+^ Tc17 cells was observed in these patients. Notably, the state of individuals before cART may impact the normalization of CD8^+^ T cell subpopulations. In this regard, 81.25% of PLWH were diagnosed at stage 2 with a short window between HIV diagnosis and treatment initiation, which is reflected by the high CD4^+^ T cell counts in these patients (≥ 500 cells/µL). Thus, this cohort comprises individuals who most likely did not reach very low CD4^+^ T cell counts, did not develop advanced disease, and had a relatively short delay in cART initiation. Therefore, it can be argued that CD8^+^ T cell disturbances are a common manifestation early after infection, both in mild and severe HIV disease, and complete functional reconstitution of this population requires long-term therapy [9, 10]. Nonetheless, a limitation of this study is the lack of data such as CD4 nadir, clinical conditions before cART initiation, or other inflammation markers that would inform on the clinical and immunological state of our cohort of individuals before cART. In this last regard, the asymptomatic reactivation of cytomegalovirus infection in PLWH has been associated with quantitative and qualitative disturbances in the pool of effector memory CD4^+^ and CD8^+^ T cells, including decreased CD4:CD8 ratios and increased frequencies of exhausted and senescent cells [28]. Furthermore, serum levels of IL-7 and IL-15 play important roles in the frequencies of both CD4^+^ and CD8^+^ T cells, as they can promote a cytotoxic and proliferative phenotype in these cells [29], and its restoration upon cART has been associated with the reconstitution of activated T cells [30]. Novel studies that address the impact of these clinical and immunological variables on the reconstitution of CD8^+^ T cell subsets in PLWH are required.

From an immunological perspective, early alterations in the intestinal barrier and microbial translocation, low-level HIV replication, concomitant coinfections, homeostatic proliferation, among other factors [31], induce an increase in inflammatory mediators such as common-γ-cytokines that may activate CD8^+^ T cells in a bystander manner [23]. The decrease in inflammatory burden is a slow and progressive process after cART initiation. The levels of systemic inflammatory mediators remain increased for a long time after viral suppression [32, 33], delaying complete CD8^+^ T cell reconstitution. Chronic CD8^+^ T cell activation/exhaustion leads to an impairment of their effector mechanisms that affect their response to new antigenic challenges. Accordingly, associations between systemic inflammation and the dysfunctional CD8^+^ T cell subsets were described before [9, 10, 34]. Interestingly, in this study, the CITRUS analysis identified a cluster of CD8^+^ T cells expressing HLA-DR, CD38, granzyme B, perforin, and CD107a that normalized in PLWH after 1 year, suggesting that this subset reached more rapid reconstitution compared to IL-17-producing cells. Considering that the success of antiretroviral therapies in developing countries such as Colombia can be significantly affected by several socio-economical barriers that can negatively affect patient adherence to the therapy, the evaluation of these cell populations by medical practitioners could represent an attractive biomarker to measure therapy efficacy, and further investigations are required in order to validate its clinical relevance. Nonetheless, a limitation of this study is the short time of follow-up, which does not allow to evaluate if other CD8^+^ T cells functional markers are reconstituted after more extended therapy.

Loss of Tc17 cells is a characteristic of progressive HIV and simian immunodeficiency virus (SIV) infections, and, similar to our results, there is limited reconstitution of this population despite cART [35, 36]. Considering that intestinal mucosa is the main niche of Tc17 cells [12], the alterations in gut epithelium and local inflammatory environment may play a role in this population’s selective lack of normalization compared with other CD8^+^ T cell effector subsets.

## Conclusions

Although the total number of CD8^+^ T cells did not show signs of reconstitution after 1 year of cART, specific qualitative features such as the activation/exhaustion phenotype and cytotoxic program of CD8^+^ T cells, but not IL-17 production, are reconstituted by continuous suppressive cART in PLWH. Although CD8^+^ T cell disturbances in HIV infection may have a common triggering mechanism, the reconstitution of CD8^+^ T cell effector subpopulations varies according to treatment time. Specifically, to the best of our knowledge, this study provides the first evidence that PLWH should undergo more than 3 years of cART in order to restore the levels of Tc17 observed in seronegative individuals. Early initiation of cART and/or adjuvant anti-inflammatory strategies could prevent or limit CD8^+^ T cell dysfunction in HIV.

## Supplementary Information

**Additional file 1: Fig. S1.** Gating strategy of CD3^+^ CD8^+^ T cells expressing CD38, HLA-DR, TIM-3 and PD-1.

**Additional file 2: Fig. S2.** Frequencies of CD8^+^ T cells that are HLA-DR^+^ CD38^+^ (A), PD-1^+^ (B), or PD-1^+^ TIM-3^+^ (C), in PLWH samples 1 and 2 classified according to the time on cART at the time of study inclusion (1–3 vs > 3 years). NS: Not statistically significant; Wilcoxon test. Patients with 1–3 years of ART = 11; patients with > 3 years of ART = 5.

**Additional file 3: Fig. S3.** Frequencies of CD8^+^ T cells that are HLA-DR^+^ CD38^+^ (A), PD-1^+^ (B), HLA-DR^+^ CD38^+^ Perforin^+^ Granzyme B^+^ (C) and HLA-DR^+^ CD38^+^ IL-17^+^ (D), in PLWH discriminated by age (≤ 35 years old vs ≥ 37 years old), at the time of inclusion (sample 1). Not statistically significant; Mann–Whitney test. Patients ≤ 35 years old = 9; patients ≥ 37 years old = 7.

**Additional file 4: Fig. S4.** Frequencies of HLA-DR^+^ CD38^+^ CD8^+^ T cells that are IFN-γ^+^ (A), and CCCL5^+^ (B), after stimulation with PMA/ionomycin, in PLWH and seronegative controls samples 1 and 2. NS: Not statistically significant. Mann–Whitney and Wilcoxon tests were used to analyze independent and paired samples, respectively. HIV samples = 16; Seronegative samples = 15. C. Frequencies of HLA-DR^+^ CD38^+^ CD8^+^ T cells that are IL-17^+^ in PLWH samples 1 and 2, discriminated by the time of therapy ($\le$ 24 months, n = 6; vs. > 25 months, n = 6); Mann–Whitney test.

**Additional file 5: Fig. S5.** Frequencies of CD8^+^ T cells that are CD107a^+^ (A), IFN-γ^+^ (B), and IL-17^+^ (C) after stimulation with HIV Gag peptides, in PLWH samples 1 and 2 classifieds according to the time on cART at the time of study inclusion (1–3 vs. > 3 years). NS: Not statistically significant; Wilcoxon test. Patients with 1–3 years of ART = 11; patients with > 3 years of ART = 5.

**Additional file 6: Fig. S6.** Frequencies of HLA-DR^+^ CD38^+^ CD8^+^ T cells that are Perforin^+^ Granzyme B^+^ (A), CD107a^+^ (B), and IL-17^+^ (C), in PLWH samples 1 and 2 classifieds according to the time on cART at the time of study inclusion (1–3 vs. > 3 years). NS: Not statistically significant; Wilcoxon test. Patients with 1–3 years of ART = 11; patients with > 3 years of ART = 3.

**Additional file 7: Table S1.** List of flow cytometry antibodies.

**Additional file 8: Table S2.** Therapeutic schemes in the study cohort.

## Data Availability

The datasets used and analyzed in the current study are available from the corresponding author on reasonable request.
