# Supplementary figures and images for: Identification of CD8+ T cell subsets that normalize in early-treated people living with HIV receiving antiretroviral therapy
Source: AIDS Res Ther. 2022 Sep 14;19:42. doi: 10.1186/s12981-022-00465-0 (PMC9476577; doi:10.1186/s12981-022-00465-0)

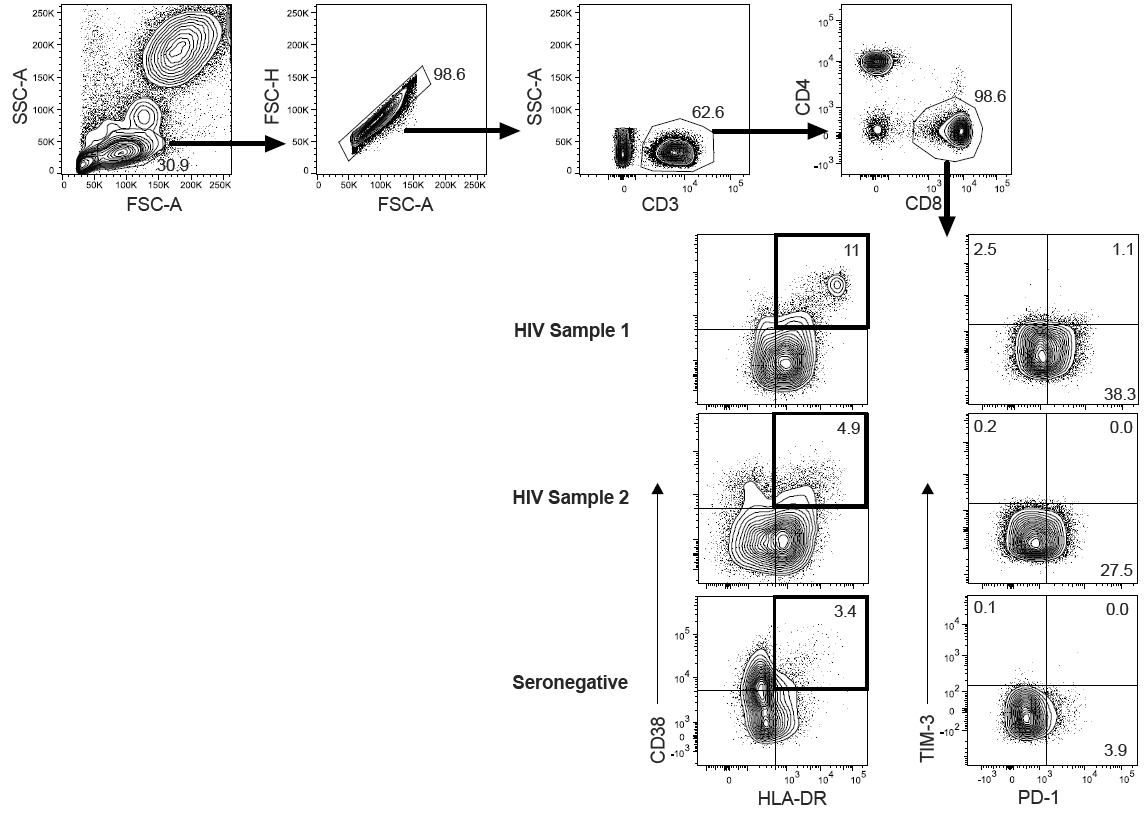

Supplement: Supplementary file 1 — Additional file 1: Fig. S1. Gating strategy of CD3+ CD8+ T cells expressing CD38, HLA-DR, TIM-3 and PD-1. [file 12981_2022_465_MOESM1_ESM.tif]
