# Supplementary material for: Identification of CD8+ T cell subsets that normalize in early-treated people living with HIV receiving antiretroviral therapy
Source: AIDS Res Ther. 2022 Sep 14;19:42. doi: 10.1186/s12981-022-00465-0 (PMC9476577; doi:10.1186/s12981-022-00465-0)
Supplement: Supplementary file 7 — Additional file 7: Table S1. List of flow cytometry antibodies. [file 12981_2022_465_MOESM7_ESM.docx]

**Identification of CD8^+^ T cell subsets that normalizes in early-treated people living with HIV receiving antiretroviral therapy**

Federico Perdomo-Celis, David Arcia-Anaya, Juan Carlos Alzate Angel, Paula A. Velilla, Francisco J Díaz, Maria Paulina Posada Vergara, María T. Rugeles, Natalia A. Taborda

**Table S1. List of flow cytometry antibodies**

| **Staining** | **Anti-human antibody** | **Clone** | **Manufacturer** |
| --- | --- | --- | --- |
| Whole blood | Anti-CD3 Alexa Fluor 700 | UCHT1 | Thermo Fisher |
|  | Anti-CD8 PE | RPA-T8 | BD |
|  | Anti-CD4 APC efluor 780 | RPA-T4 | Thermo Fisher |
|  | Anti-HLA-DR FITC | LN3 | Thermo Fisher |
|  | Anti-CD38 PE Cy7 | HIT2 | Thermo Fisher |
|  | Anti-PD-1 PerCP efluor 710 | J105 | Thermo Fisher |
|  | Anti-TIM3 efluor 450 | F38 2E2 | Thermo Fisher |
| Cultured PBMC | Anti-CD3 PerCP | SK7 | BD |
|  | Anti-CD8 Alexa Fluor 700 | OKT8 | Thermo Fisher |
|  | Anti-HLA-DR APC efluor 780 | LN3 | Thermo Fisher |
|  | Anti-CD38 PE efluor 610 | HIT2 | Thermo Fisher |
|  | Anti-CD107a | H4A3 | BD |
|  | Anti-Perforin PE | D48 | Biolegend |
|  | Anti-Granzyme B FITC | GB11 | BD |
|  | Anti-IFN-γ PE Cy7 | 4S.B3 | Thermo Fisher |
|  | Anti-IL-17A BV510 | N49-653 | BD |
|  | Anti-CCL5 BV421 | 2D5 | BD |
