# Supplementary material for: Identification of CD8+ T cell subsets that normalize in early-treated people living with HIV receiving antiretroviral therapy
Source: AIDS Res Ther. 2022 Sep 14;19:42. doi: 10.1186/s12981-022-00465-0 (PMC9476577; doi:10.1186/s12981-022-00465-0)
Supplement: Supplementary file 8 — Additional file 8: Table S2. Therapeutic schemes in the study cohort. [file 12981_2022_465_MOESM8_ESM.docx]

**Identification of CD8^+^ T cell subsets that normalizes in early-treated people living with HIV receiving antiretroviral therapy**

Federico Perdomo-Celis, David Arcia-Anaya, Juan Carlos Alzate Angel, Paula A. Velilla, Francisco J Díaz, Maria Paulina Posada Vergara, María T. Rugeles, Natalia A. Taborda

**Table S2. Therapeutic** **schemes in the study cohort**

| **ART Combination** | **Number of treated patients** | **Percentage of treated patients** |
| --- | --- | --- |
| Abacavir/Lamivudine/Efavirenz | 5 | 31.25 |
| Emtricitabine/Tenofovir/Efavirenz | 9 | 56.25 |
| Raltegravir/Tenofovir/Emtricitrabine | 2 | 12.5 |
